# L-(+)-Tryptophan methyl ester derived polymeric microbeads as an efficient heterogeneous catalyst for green synthesis of 2-amino-4-(nitromethyl)-4*H*-chromene-3-carbonitriles

**DOI:** 10.55730/1300-0527.3467

**Published:** 2022-07-19

**Authors:** Ayşe HALIÇ POSLU, Bilgen OSMAN, Yunus KAYA, Ömer KOZ, Gamze KOZ

**Affiliations:** 1 Department of Chemistry, Faculty of Engineering and Natural Sciences, Bursa Technical University, Bursa, Turkey; 2 Department of Chemistry, Faculty of Arts and Science, Bursa Uludağ University, Bursa, Turkey

**Keywords:** Polymeric microbeads, L-(+)-tryptophan methyl ester, nitromethane addition, π-π interaction, DFT calculation

## Abstract

The cross-linked microbeads with average diameter of 106–300 μm, [poly(EGDMA-MATrp)], were obtained by copolymerization reaction of N-methacryloyl-L-(+)-tryptophan methyl ester (MATrp) with ethylene glycol dimethacrylate (EGDMA) and successfully applied as a heterogeneous catalyst in conjugate addition reaction of nitromethane to substituted 2-iminochromenes in aqueous media. A variety of 2-amino-4-(nitromethyl)-4*H*-chromene-3-carbonitriles has been synthesized in good yields. Polymeric microbeads were very durable and reused 5 times without a significant loss of activity. DFT calculations and experimental results revealed the significant role of π-π interactions as well as hydrogen bonding in the reaction mechanism.

## 1. Introduction

Functional polymeric microbeads have found many practical applications in chemical separation and purification, wastewater treatment, bioengineering, materials science, drug delivery systems, and cancer therapy [1–6]. The development of polymeric microbeads as heterogeneous organocatalysts has attracted increasing attention due to the advantages such as easy product isolation and catalyst reusability. Polymeric microbeads have been successfully applied to a variety of organic reactions such as Suzuki Miyaura reaction [7], asymmetric Michael, aldol reactions [8], esterification [9], substitution [10] and A^3^ coupling reactions, which is a three-component coupling of aldehyde, amine and alkyne for the synthesis of propargylamines [11]. For example, cinchonidinium salts of core-corona polymer microspheres were applied several times in asymmetric alkylations without loss of the enantioselectivity [12]. Shi and coworkers reported the synthesis and characterization of a polymer microsphere catalyst [13]. Ionic liquid immobilized catalyst was applied in the Knoevenagel condensation of ethyl cyanoacetate and benzaldehyde. The benzaldehyde conversion was maintained at 92% after six times reuse. Polymeric microbeads have a great application potential in many other organic reactions as catalysts because of their mechanical strength and high surface area. The additional advantage of polymeric microbeads is the ability to functionalize their surface area with hydrophobic and hydrophilic groups, which play an active role in catalytic transition states.

Osman et al. used N-methacryloyl-L-(+)-tryptophan methyl ester (MATrp) monomer to prepare polymeric microbeads for removal [14, 15] and solid phase extraction [16–18] of some aromatic organic contaminants from aqueous solutions. Poly(ethylene glycol dimethacrylate N-methacryloyl-L-(+)-tryptophan methyl ester) [poly(EGDMA-MATrp)] microbeads were effectively used to remove diethyl phthalate from aqueous solution [19]. The results of these studies showed that the microbeads have a high affinity for aromatic compounds owing to hydrophobic interactions such as π-π stacking between MATrp residues of cross-linked polymer and target analytes. The importance of π-π interactions in transition states (TS) of the catalytic reactions have been emphasized in some recent studies [20–24]. As noncovalent interactions have been the main forces effective on catalytic transition states, we decided to investigate the efficiency of [poly(EGDMA-MATrp)] polymeric microbeads in an organic reaction containing hydrophobic substrates such as chromenes and reveal a mechanistic explanation including π-π interactions.

Chromenes are an important class of heterocyclic compounds, widely distributed in natural products. They possess a wide range of biological activities such as anticancer [25], antimicrobial and antifungal [26], antioxidant [27] and anti-HIV [28]. A number of methods using homogeneous [29–31] and heterogeneous catalysts [32–36] have been developed to synthesize functionalized 4*H*-chromenes owing to their importance. We have reported an enantioselective method using a Schiff base-Cu(II) catalyst [37] and a Lewis base catalyzed method for the synthesis of rasemic 2-amino-4-(nitromethyl)-4*H*-chromene-3-carbonitriles from 2-iminochromenes [38]. However, this is the first report on the use of a heterogeneous catalyst in nitromethane additions to 2-iminochromenes. We also present the crucial role of π-π interactions in transition state of the reaction depending on both computational and experimental results.

## 2. Materials and Methods

All solvents/reagents were obtained commercially from Fluka and Sigma Aldrich and used as purchased. Silica gel F254 (Merck 5554) precoated plates were used for monitoring. A 400 MHz Bruker NMR spectrometer and a Thermo-Nicolet 6700 FT-IR spectrometer was used for NMR and FT-IR analysis, respectively. Polymeric microbeads were analyzed with scanning electron microscopy (Carl Zeiss/Gemini 300). Melting points (mp) were recorded with an electro thermal digital mp apparatus. 2-iminochromenes (**1a**-**m**) were synthesized by a pyrrolidine catalyzed method and known compounds were characterized according to reported mp and 1H NMR data [37–40]. The 6-I substituted 2-iminochromene (**1l**) was not stable at room temperature so it was used as crude in the addition reaction without isolation but the addition product (**2l**) was isolated and fully characterized.

### 2.1. Synthesis of [Poly(EGDMA-MATrp)] microbeads

The suspension polymerization technique was used to prepare [Poly(EGDMA-MATrp)] microbeads as described in our previous report [14]. EGDMA and MATrp were used as a cross-linker and a monomer, respectively. Polymerization mixture was prepared by dispersing the organic phase including EGDMA (5 mL), MATrp (4 mL), and toluene (10 mL) in aqueous phase prepared via dissolution of 200 mg poly(vinyl alcohol) (PVA) in deionized water (50 mL). After addition of 2,2′-azobisisobutyronitrile (AIBN) (100 mg), the mixture was polymerized at 85 °C with a 600 rpm stirring rate for 8 h. The unreacted residues were washed with excess amount of water and ethanol. The resulted microbeads were dried in a vacuum oven at 50 °C. A Carl Zeiss/Gemini 300 scanning electron microscope was used to monitor the physical morphology of the microbeads.

### 2.2. Synthesis of 2-iminochromenes (1a-m)

Malononitrile (5 mmol) and the corresponding aromatic aldehyde (5 mmol) were dissolved in a mixture of MeOH:H_2_O (3:1) (4 mL) and a catalytic amount of pyrrolidine (0.75 mmol) was added to the mixture. The product precipitated and filtered, washed with 3:1 MeOH:H_2_O and dried in a vacuum oven.

#### 2-Imino-8-nitro-2H-chromene-3-carbonitrile (**1i**, C_10_H_5_N_3_O_3_)

Yield 56%; mp 204 °C (decomp.); R*_f_* = 0.48 (ethyl acetate/hexane, 1:1); 1H NMR (400 MHz, DMSO-d_6_): δ (ppm) 9.37 (bs, 1H, NH), 8.47 (s, 1H, H4), 8.20–8.17 (dd, *J_1_* = 1.2, *J_2_* = 8.0 Hz, 1H, ArH), 7.90–7.88 (dd, *J_1_* = 1.6, *J_2_* = 8.0 Hz, 1H, ArH), 7.42 (t, *J* = 8.0 Hz, 1H, ArH). 13C NMR (DMSO-d_6_, 100 MHz): δ (ppm) 161.2, 146.7, 146.4, 144.9, 141.6, 134.9, 124.6, 124.4, 120.5, 105.9. FT-IR (neat): *ν̄* = 3270, 2238, 1662, 1519, 1231 cm^−1^. Anal. Calcd. for: C_10_H_5_N_3_O_3_ (%):C, 55.82; H, 2.34; N, 19.53; Found: C, 56.73; H, 2.24; N, 19.78.

#### 8-Bromo-2-imino-2H-chromene-3-carbonitrile (**1j**, C_10_H_5_BrN_2_O )

Yield 50%; mp 170 °C (decomp.); R*_f_* = 0.53 (ethyl acetate/hexane, 1:2); 1H NMR (400 MHz, DMSO-d_6_): δ (ppm) 9.14 (bs, 1H, NH), 8.38 (s, 1H, H4), 7.87–7.85 (dd, *J_1_* = 1.6, *J_2_ =* 8.0 Hz, 1H, ArH), 7.60–7.58 (dd, *J_1_* = 1.6, *J_2_* = 7.6 Hz, 1H, ArH), 7.20 (t, *J* = 7.6 Hz, 1H, ArH). 13C NMR (DMSO-d_6_, 100 MHz): δ (ppm) 150.4, 150.3, 146.5, 137.0, 129.1, 125.4, 119.1, 114.9, 108.3, 105.2. FT-IR (neat): *ν̄*= 3292, 3231, 2227, 1663, 1593, 1199 cm^−1^. Anal. Calcd. for: C_10_H_5_BrN_2_O (%):C, 48.22; H, 2.02; Br, 32.08; N, 11.25, Found: C, 49.56; H, 2.06; N, 10.97.

### 2.3. Synthesis of 2-amino-4-(nitromethyl)-4*H*-chromene-3-carbonitriles (2a-m)

[Poly(EGDMA-MATrp)] microbeads (5mg) and the corresponding 2-iminochromene (**1a-m**) (0.25 mmol) were added to the solution of nitromethane (0.75 mmol) in 5:1 methanol:H_2_O (1 mL). The mixture was stirred at room temperature until the corresponding 2-iminochromene was consumed. Methanol was removed by rotary evaporator and the crude product was extracted to ethyl acetate. The organic phase was dried over Na_2_SO_4_, filtered, and concentrated under reduced pressure to afford the crude product. Column chromatography was used to purify the crude product. The previously reported 4*H*-chromene-3-carbonitriles were characterized using 1H NMR literature data and melting points [37–43].

#### 2-Amino-8-hydroxy-4-(nitromethyl)-4H-chromene-3-carbonitrile (**2e**, C_11_H_9_N_3_O_4_)

Yield 51%; mp 173 °C; R*_f_* = 0.43 (ethyl acetate/hexane, 1:1); 1H NMR (400 MHz, DMSO-d_6_): δ(ppm) 9.81 (bs, 1H, OH), 7.06 (bs, 2H, NH_2_), 6.94 (t, *J* = 7.6 Hz, 1H, ArH), 6.84–6.81 (dd, *J_1_* = 1.2, *J_2_* = 8.0 Hz, 1H, ArH), 6.70–6.68 (dd, *J_1_* = 0.8, *J_2_* = 7.6 Hz, 1H, ArH), 4.74–4.70 (dd, *J_1_* = 5.6, *J_2_* = 12.4 Hz, 1H, CH_2_NO_2_), 4.63–4.59 (dd, *J_1_* = 5.6, *J_2_* = 12.0 Hz, 1H, CH_2_NO_2_), 4.24 (t, *J* = 5.6 Hz, 1H, H4). 13C NMR (100 MHz, DMSO-d_6_): δ(ppm) 162.3, 145.2, 138.1, 124.5, 120.6, 119.9, 117.6, 115.5, 80.8, 50.0, 35.0. FT-IR (neat): *ν̄* = 3473, 3361, 3206, 2189, 1549 cm^−1^. Anal. Calcd. for: C_11_H_9_N_3_O_4_ (%):C, 53.44; H, 3.67; N, 17.00; Found: C, 53.61; H, 3.68; N, 15.91.

#### 2-amino-8-nitro-4-(nitromethyl)-4H-chromene-3-carbonitrile (**2i**, C_11_H_8_N_4_O_5_)

Yield 31%; mp 152–154 °C; R*_f_* = 0.65 (ethyl acetate/hexane, 1:1); 1H NMR (400 MHz, DMSO-d_6_): δ(ppm) 7.94–7.92 (dd, *J_1_* = 1.6, *J_2_* = 8.4 Hz, 1H, ArH), 7.71–7.69 (dd, *J_1_* = 1.2, *J_2_* = 8.0 Hz, 1H, ArH), 7.41 (bs, 2H, NH_2_), 7.38 (d, *J* = 8.0 Hz, 1H, ArH), 4.92–4.87 (dd, *J_1_* = 4.8, *J_2_* = 12.8 Hz, 1H, CH_2_NO_2_), 4.77–4.72 (dd, *J_1_* = 4.8, *J_2_* = 12.8 Hz, 1H, CH_2_NO_2_), 4.45 (t, *J* = 4.8 Hz, 1H, H4). 13C NMR (400 MHz, DMSO-d_6_): δ(ppm) 161.1, 141.8, 138.2, 133.2, 124.6, 124.4, 122.5, 118.9, 80.3, 50.3, 34.3. FT-IR (neat): *ν̄* = 3421, 3327, 2190, 1645, 1519 cm^−1^. Anal. Calcd. for: C_11_H_8_N_4_O_5_ (%):C, 47.83; H, 2.92; N, 20.28, Found: C, 47.18; H, 2.81; N, 18.55.

#### 2-Amino-6-iodo-4-(nitromethyl)-4H-chromene-3-carbonitrile (**2l**, C_11_H_8_IN_3_O_3_)

Yield 38%; mp 196 °C; R*_f_* = 0.35 (ethyl acetate/hexane, 1:2); 1H NMR (400 MHz, CDCl_3_): δ(ppm) 7.62–7.60 (dd, *J_1_* = 2.0, *J_2_* = 8.4 Hz, 1H, ArH), 7.49 (d, *J* = 2.0 Hz, 1H, ArH), 6.80 (d, *J* = 8.8 Hz, 1H, ArH), 4.83 (bs, 2H, NH_2_), 4.62–4.58 (dd, *J_1_* = 4.8, *J_2_* = 12.4 Hz, 1H, CH_2_NO_2_), 4.55–4.50 (dd, *J_1_* = 6.4, *J_2_* = 12.4 Hz, 1H, CH_2_NO_2_), 4.30–4.27 (dd, *J_1_* = 4.8, *J_2_* = 6.4 Hz, 1H, H4). 13C NMR (100 MHz, CDCl_3_): δ(ppm) 161.8, 149.5, 138.5, 136.6, 121.4, 118.9, 118.7, 88.4, 79.9, 53.2, 34.4. FT-IR (neat): *ν̄* = 3442, 3325, 3209, 2918, 2202, 1649, 1531 cm^−1^. Anal. Calcd. for: C_11_H_8_IN_3_O_3_ (%):C, 37.00; H, 2.26; N, 11.77, Found: C, 37.16; H, 1.98; N, 11.21.

### 2.4. DFT calculations

Density functional theory (DFT) with the wB97X-D method as applied in the GAUSSIAN 09 program package was conducted for the calculations. Dispersion correction for energy barrier and reaction heat were estimated with the wB97X-D method reported by Grimme [44], as DFT poorly describes dispersion effects. All calculations and harmonic frequencies to find transition states (one imaginary force constant only) or local minima (all positive force constants) of the structures were calculated with the 6-311++G(d,p) basis set in the gas phase.

## 3. Results and discussion

**Figure 1:**
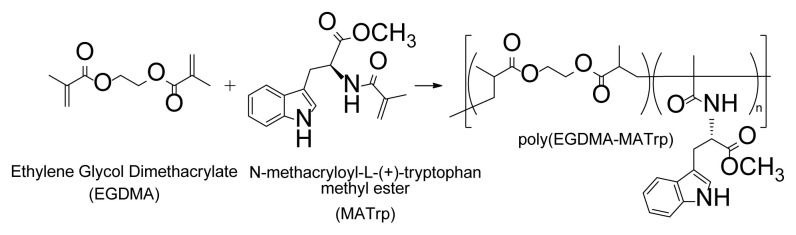
Preparation of poly(EGDMA-MATrp) microbeads.

The [poly(EGDMA-MATrp)] microbeads were synthesized according to the literature (Figure 1) [14].

**Figure 2:**
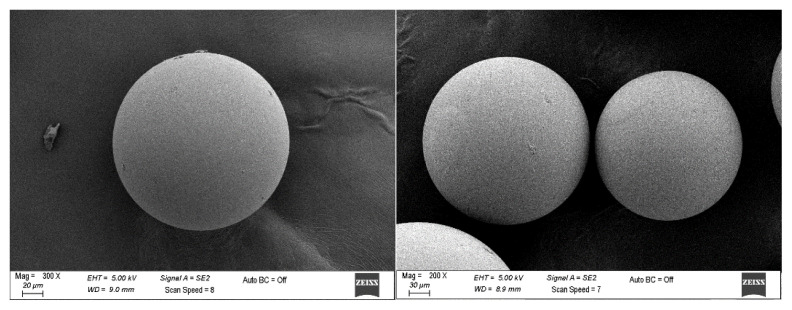
SEM images of [poly(EGDMA-MATrp)] microbeads.

The cross-linked microbeads were obtained in a spherical form with the size range of 106–300 μm in diameter. SEM images of the microbeads are shown in Figure 2.

The [poly(EGDMA-MATrp)] microbeads were used as the heterogeneous catalyst in the model reaction between nitromethane and **1a** (Table 1).

The best results were obtained in aqueous media (entry 4, 5) and the reaction yields were very poor in polar aprotic solvents (entry 6–8) and toluene. The catalyst loading was examined in water and 90% yield was obtained with 5 mg use of [poly(EGDMA-MATrp)] microbeads (entry 4). Then we investigated the substrate scope of the reaction. As the yields of the first experiments performed with substituted 2-iminochromenes in water were moderate, we carried out the reactions in a mixture of methanol:water (5:1) (Table 2).

A series of 2-amino-4-(nitromethyl)-4*H*-chromene-3-carbonitrile (**2a-m**) was synthesized with moderate to good yields (31–95 %) with both, electron-donating and electron-withdrawing substituents on the aromatic ring. In general, reactions of the substrates with electron-donating substituents were high yielding in short reaction times (**2c**, **2d**, **2g**, **2h**) while the electron-withdrawing substrates dramatically decreased the reaction rate (**2i**). These results were attributed to the significant role of π-π interactions in aqueous media between substrates and catalyst as the electron withdrawing substituents weaken π stacking interactions by decreasing the pi electron density of the aromatic ring. Regardless of inductive effects in substituted aromatic rings of 2-iminochromenes, the position of the substituents also affected the reaction yields. In general, higher yields were obtained with 6-substituted (**2b**-**d**, **2f**, **2k**) and 7-substituted (**2h**) 2-iminochromenes while lower yields were obtained with 8-substituted 2-iminochromenes (**2e**, **2g**, **2i**, **2j**).

**Figure 3:**
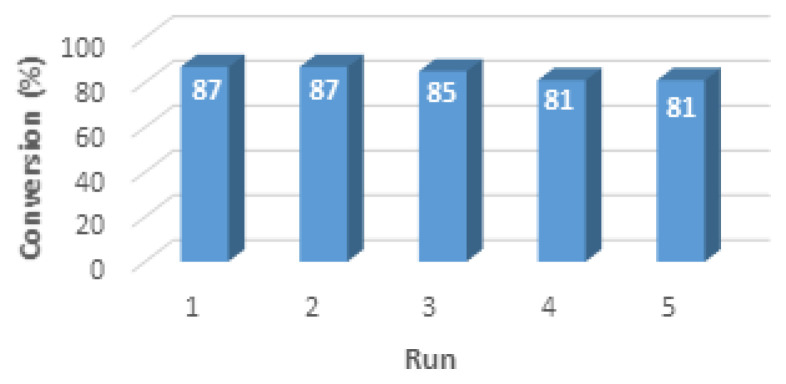
Catalyst reuse; reaction conditions: **1a** (0.25 mmol), nitromethane (0.75 mmol), [poly(EGDMA-MATrp)] microbeads (5 mg), MeOH:H_2_O (5:1) (1 mL).

Finally, we tested the reusability of the catalyst in the model reaction between nitromethane and **1a** (Figure 3). The catalyst was centrifuged and separated from reaction medium, washed several times with methanol and air dried after each use.

A distinct decrease was not observed in the activity of the catalyst. The final yield of **2a** decreased only from 87% to 81% at the end of fifth reaction run.

**Figure 4:**
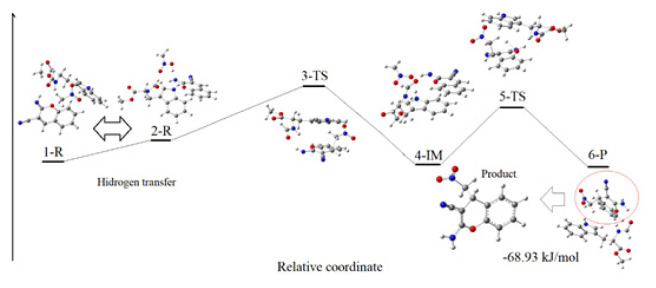
Potential energy diagram & geometry transformations between compound 1-R and compound 6-P, optimized geometries of the reactants, IN, TS, and product calculated by WB97XD/6-311G++(d,p).

We also performed DFT calculations and suggested a transition state (TS) including all noncovalent interactions. The related potential energy surfaces (PES’s) and the optimized structures of the intermediates (IM) and TS along the reaction pathway are shown in Figure 4, while the relative energies of the reactants, IM, TS, and product are given in Table 3.

When the optimized molecular structures are examined, it is observed that the catalyst coordinates to both 2-iminochromene and nitromethane molecules through hydrogen bonding. The formation of intermediate 4-IM via nucleophilic attack of nitromethane to 2-iminochromene substrate is the first step of the mechanism. The transition state (3-TS) formed in this step determines the rate of the reaction and plays a key role in the mechanism.

**Figure 5:**
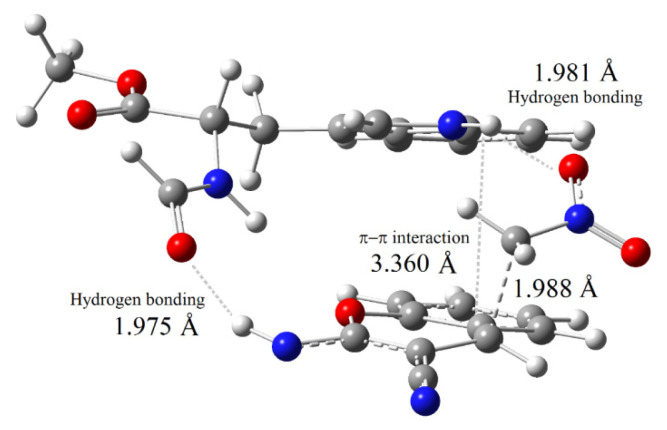
Optimized structure and intermolecular interactions (<3.5 Å) of the 3-TS complex molecule.

The optimized structure of the 3-TS molecule in the rate-determining step of the reaction is depicted in detail in Figure 5. The catalyst molecule coexists with the 2-iminochromene with a NH ···· O (catalyst) hydrogen bond with a length of 1.975 Å, while it forms a hydrogen bond between the nitromethane molecule and the NH ···· O (nitromethane) atoms (1.981 Å length) on the other hand. In addition, π-π interactions at a distance of 3.360 Å between the aromatic rings of the catalyst and the 2-iminochromene molecules play an important role in the stability of the transition state. The energy barrier (*E*_a_) of this step is 63.26 kJmol^−1^. The distance between C4 carbon atom of 2-iminochromene and carbon atom of nitromethane is calculated as 1.998 Å. The calculation of the single negative frequency at −548 cm^−1^ in 3-TS supports the correct optimization of the molecule. The second step of the reaction is the proton transfer step, and it takes place rapidly over 5-TS. The energy barrier in this step is calculated to be 48.99 kJmol^−1^.

When the reaction mechanism is considered as a whole, the formation energy of the product 6-P is calculated as −68.93 kJmol^−1^.

In conclusion, cross-linked microbeads, [poly(EGDMA-MATrp)], were successfully applied as a heterogeneous catalyst in conjugate addition reactions of nitromethane to substituted 2-iminochromenes. Experimental results revealed the activating role of hydrophobic interactions in aqueous reaction medium. We obtained the best results with 2-iminochromene substrates having electron rich aromatic rings that is able to form stronger stacking interactions with the catalyst. DFT calculations also revealed the significant role of π-π interactions as well as hydrogen bonding in the reaction mechanism. Polymeric microbeads were very durable and reused 5 times without a significant loss of activity. The design and synthesis of polymeric microbeads with more effective chiral microenvironments for asymmetric synthesis is under investigation.

## Figures and Tables

**Table 1:** [Poly(EGDMA-MATrp)] catalyzed synthesis of 2-amino-4-(nitromethyl)-4*H*-chromene-3-carbonitriles **2a**^a^. Screening of solvents and catalyst loading.

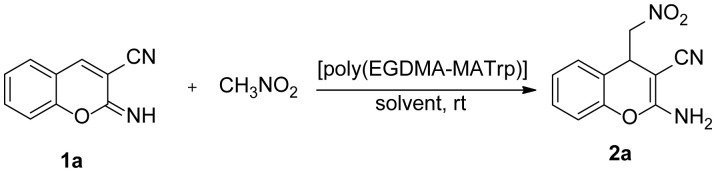			
Entry	Solvent	Time (h)	Yield (%)^b^
1	methanol	18	68
2	ethanol	24	38
3	2-propanol	24	35
4	water	39	90
5	methanol:water (5:1)	24	87
6	chloroform	96	trace
7	acetone	96	trace
8	tetrahydrofuran	96	trace
9	toluene	96	trace
10^c^	water	100	65
11^d^	water	96	30
12^e^	water	120	-

a Reaction conditions: 1a (0.25 mmol), nitromethane (0.75 mmol), [poly(EGDMA-MATrp)] (5mg), solvent (1 cm^3^).

b Isolated yields after column chromatography.

c 1 mg catalyst loading.

d 10 mg catalyst loading.

e without catalyst.

**Table 2:**
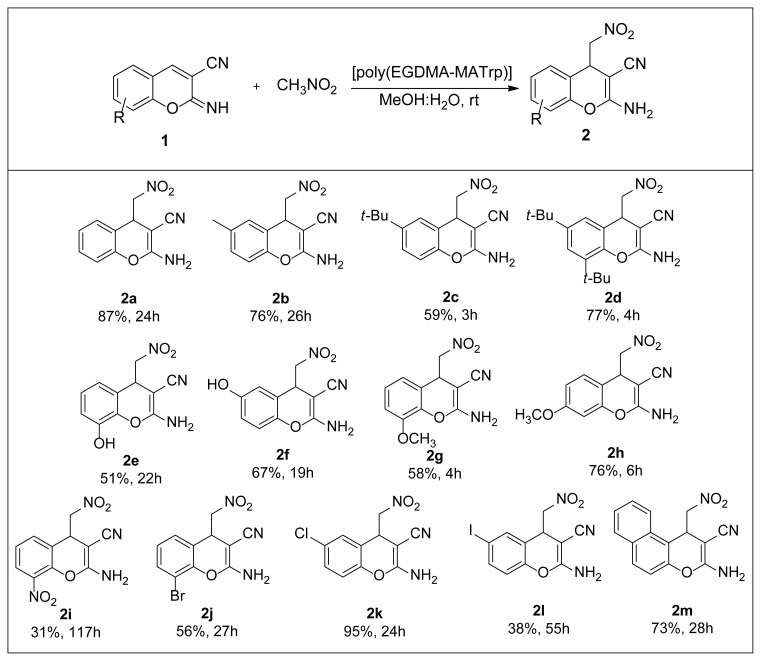
Conjugate addition of nitromethane to 2-iminochromenes^a^. Substrate scope.

a Reaction conditions: **1a** (0.25 mmol), nitromethane (0.75 mmol), [poly(EGDMA-MATrp)] (5mg), MeOH:H_2_O (5:1) (1 cm^3^).

b Isolated yields after column chromatography.

**Table 3:** Negative frequency and relative energy values calculated by WB97XD/6-311G++(d,p) level.

Species	Energy (a.u.)	Relative energy (kJ/mol)	Negative frequency (cm^−1^)
1-R	−1653.29621195	0.00	-
2-R	−1653.28266504	35.56	-
3-TS	−1653.27211882	63.26	−548
4-IM	−1653.28994310	16.45	-
5-TS	−1653.27755360	48.99	−1217
6-P	−1653.32244695	−68.93	-
